# Wind energy resource assessment and wind turbine selection analysis for sustainable energy production

**DOI:** 10.1038/s41598-024-61350-6

**Published:** 2024-05-10

**Authors:** Paraschiv Spiru, Paraschiv Lizica Simona

**Affiliations:** Dunarea de Jos University of Galati, 800008 Galati, Romania

**Keywords:** Wind energy, Wind resource assessment, Renewable energy analysis, Wind power density, Sustainable energy production, CO_2_ emission avoided, Carbon dioxide, Climate sciences, Ecology, Environmental sciences, Energy science and technology

## Abstract

The objective of this study is to perform an analysis to determine the most suitable type of wind turbine that can be installed at a specific location for electricity generation, using annual measurements of wind characteristics and meteorological parameters. Wind potential analysis has shown that the analyzed location is suitable for the development of a wind farm. The analysis was carried out for six different types of wind turbines, with a power ranging from 1.5 to 3.0 MW and a hub height set at 80 m. Wind power potential was assessed using the Weibull analysis. The values of the scale coefficient c were determined, and a large monthly variation was observed, with values ranging from 1.92 to 8.36 m/s and an annual value of 4.95 m/s. Monthly values for the shape coefficient k varied between 0.86 and 1.53, with an annual value of 1.07. Additionally, the capacity factor of the turbines was determined, ranging from 17.75 to 22.22%. The Vestas turbine, with a nominal power of 2 MW and a capacity factor of 22.22%, proved to be the most efficient wind turbine for the specific conditions of the location. The quantity of greenhouse gas emissions that will be reduced if this type of turbine is implemented was also calculated, considering the average CO_2_ emission intensity factor (kg CO_2_/kWh) of the national electricity system.

## Introduction

Renewable energy has increasingly gained importance to reduce pollution, CO_2_ emissions, mitigate climate change, and promote sustainable development^1^.

The world is undergoing an energy transition to limit climate change, with the main basis of this transition being the acceleration of clean energy use^2^. The Intergovernmental Panel on Climate Change (IPCC) has highlighted the need for immediate action to limit the increase in the global average temperature to 1.5 °C. The total annual greenhouse gas emissions, approximately 14.6 Gt CO_2eq_ can be attributed to coal burning, making it responsible for about 30% of total greenhouse gas emissions^3^. Figure 2 displays the monthly average carbon dioxide concentration starting from 2000. It can be observed that there was an increase of 5.27% between 2000 and 2010, 11.95% between 2000 and 2020, and 14.44% until January 2024. Renewable energy sources represent a significant alternative to burning fossil fuels. The International Energy Agency emphasizes that the development of hydro, wind, and solar energy is crucial for achieving the global goal of "net zero" greenhouse gas emissions^4^. The amount of electricity produced from these renewable sources largely depends on weather conditions. Changes in wind speed and direction affect the continuity of energy generation in wind farms.

Generating renewable energy from wind is among the most effective ways to reduce carbon emissions and achieve carbon neutrality^5^. However, the wind resource is susceptible to be impacted by climate change, as global temperature rise will reshape patterns of atmospheric circulation^6^. Due to the evolution of global temperatures and atmospheric movement patterns under climate variability, significant changes may occur in the spatial and temporal distributions of wind resources in the future.

The assessment of wind energy requires data collection and the use of analytical methods and techniques to estimate the availability of winds for a wind turbine over its lifetime^7^. Information about wind availability is essential for determining how much energy the wind energy farm will produce, to define its mode of operation, and ultimately, to estimate its economic viability. Therefore, the use of successful forecasting models is vital to provide useful predictions regarding the wind energy potential of interest for the long-term development of wind farms^8^.

Before installing a wind turbine, the measurement and analysis of wind resources must be carried out to assess the potential for wind energy generation and to select the appropriate wind turbine model^9,10^. The power produced by a wind turbine varies considerably depending on the distribution of wind speed, even if the average wind speed is the same. This is because wind energy is determined by the cube of the wind speed, while the average wind speed is determined by the arithmetic mean. Generally, wind resources must be measured for at least one year (Kose, 2004; Kang et al., 2021), but there are several limitations, including cost-related issues, such as site selection, installation of weather masts or LiDAR after physical and geotechnical ocean studies, and additional maintenance^11,12^.

**Figure 1:**
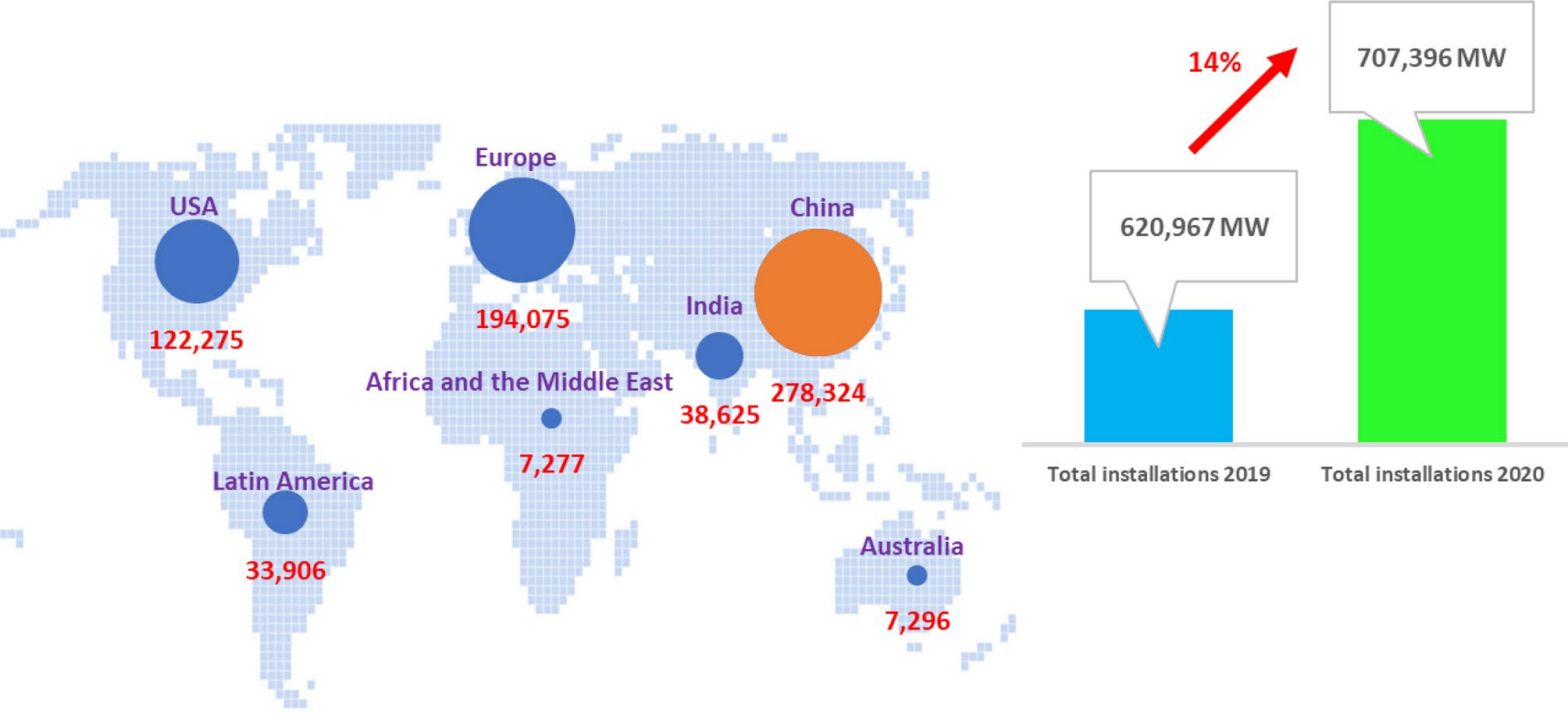
Global distribution of onshore wind power in 2021 (Python 3.11, https://www.python.org).

**Figure 2:**
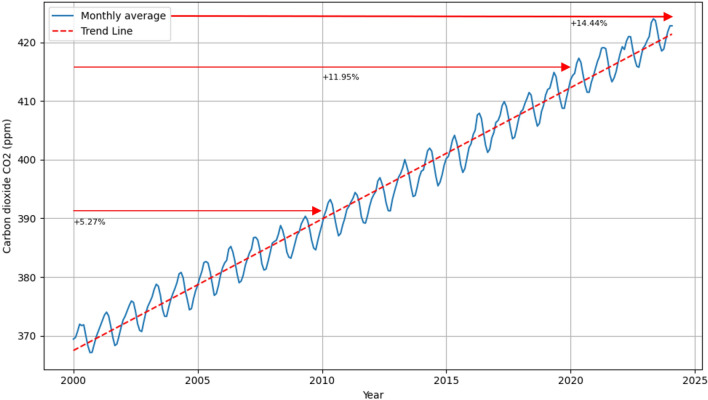
Global average of atmospheric carbon dioxide (ppm)^14–17^ (Python 3.11, https://www.python.org).

Wind energy stands out as one of the rapidly growing renewable energy sources in recent years. According to the 2021 Global Wind Energy Statistics and as shown in Fig. 1, the countries with the highest onshore wind energy capacity by the end of 2021 were China, with 39.34% (278,324 MW), followed by the United States with 17.28% (122,275 MW), and Europe with 27.43% (194,075 MW) of the total installed capacity^13^. Figure 1 shows that the global total onshore wind energy capacity installed in 2021 increased by 14% compared to the capacity installed in 2020 (Fig. 2).

As a result, accurately estimating the wind potential for a specific location and selecting the optimal type of wind turbine for installation are critical analyses in wind farm development^18,19^. Following an assessment of wind potential at a specific location, the process of selecting the most efficient turbine for installation becomes crucial. An incorrect selection could lead to inefficient financial investment due to energy underproduction^20^. Various methods have been employed by different authors to address the issue of choosing the optimal wind turbine for a specific location, utilizing the wind turbine capacity factor^21^. Waldemar Kuczynski and colleagues conducted a study for two locations in Poland to select an optimal wind turbine based on annual wind measurements and various nominal turbine powers^22^.

Regarding wind speed measurement data, Rajat Kanti Samal confirmed the importance of meteorological data measured at the analyzed location for wind resource assessment, demonstrating that wind energy density calculated using NASA MERRA-2 satellite data showed significant differences in hourly, monthly, and seasonal variations and presented better correlations only for longer periods of at least one year^23^.

Many studies have been conducted to determine wind potential; however, no method can precisely predict wind potential in any location due to the highly variable nature of wind speed, influenced by variable meteorological factors specific to each site.

The evaluation of wind potential at a specific location is a multi-stage analytical and assessment process. In general, there is no one, precise method for assessing wind potential at a location as it depends on a variety of factors and variables. The most commonly methods and techniques for assessing wind potential include the analysis of on-site measured meteorological data, GIS analysis, and machine learning.

Geographic Information Systems (GIS) are valuable tools for analyzing and assessing wind energy potential. However, they have some limits in wind energy applications. One limitation is the accuracy of the data used, meteorological data precision may vary depending on the data source and quality. Inaccurate data can lead to errors in wind resource assessments. For example, Cavazzi and Dutton used offshore wind speed data from the Renewable Energy Atlas, ABP mer, but the errors ranging from − 5 to 29% between long-term measurements and the proposed model^24,25^. This introduced both underestimating and overestimation of wind speed at the location [source].

Another limitation of GIS-based wind resource assessments is the modeling assumptions, such as wind speed extrapolation from meteorological data or simplification of atmospheric conditions. These assumptions may not always accurately reflect the true wind characteristics of a site.

Temporal variability is another limitation due to GIS data often representing static snapshots of geographic features and meteorological conditions at specific time points. Wind energy applications require consideration of temporal variability, such as diurnal and seasonal changes in wind speed and direction. Complex terrain can also introduce limitations as features like hills, valleys, and forests can significantly influence local wind patterns. Simplified terrain modeling may lead to underestimation or overestimation of wind resources, and interpolation in areas with limited available meteorological data over a large area may introduce high uncertainties.

Machine learning (ML) has shown promise in wind resource assessment, but it comes also with some limitations. ML algorithms require large volumes of high-quality data for training, validation, and testing. ML algorithms, such as deep learning models, are considered black-box models, making it challenging to interpret their predictions and understand the underlying factors influencing the outcomes. ML models trained on specific geographic locations may struggle to generalize conditions to new locations. Factors like terrain variations, climate, and meteorological parameters can significantly impact the performance and generalization capability of ML models. Additionally, ML models inherently introduce uncertainties and can suffer from overfitting and underfitting.

Li and others have observed that the Extreme Learning Machine (ELM) does not perform well for short-term forecasting, with an error of 21.09% for a forecast of 66 days, and for ultra-short-term forecasting after error correction, the total error was 5.76%^26^.

Despite the limitations, GIS and ML remain useful tools for conducting preliminary site assessments to identify wind potential. Due to the limitations and uncertainties associated with GIS and ML data and models, they should be complemented with ground-based measurements and field assessments to obtain more precise and reliable results.

Together, on-site meteorological parameter measurements, simulations, and statistical analyses significantly improve wind energy predictions.

Wind potential analysis is a complex task as air flow is influenced by a multitude of factors such as aerosols, clouds, humidity, energy exchange between the Earth's surface and the atmosphere, and terrain characteristics. Wind is thus a variable and uncertain energy source, dependent on a range of complex atmospheric and topographic conditions. Many wind farms generate less energy than expected due to uncertainties in wind forecasting and in simulating the complex flows within wind turbine farms^27^.

In this study, due to the availability of hourly measured meteorological data at the location, we conducted a detailed analysis of wind potential in the area. This analysis included essential parameters such as wind speed and direction, air temperature, relative humidity, and other relevant meteorological parameters. With the help of these precise and up-to-date data, we were able to determine the characteristics and variability of the wind regime in the studied area, as well as the wind potential of the area, including estimates of wind energy production. The availability of hourly measured meteorological data at the location was essential for conducting a comprehensive and accurate analysis of wind potential in the area.

## Methodologies

### Site meteorological data

The analysis was conducted for a location in southeastern Romania, in Tulcea County, at 45.27° N and 28.42° E. The data measured for the analysis included wind speed, wind direction, temperature, and air pressure^28^.

The distance between the measuring station and the analyzed location is approximately 500 m. Since the terrain has the same characteristics and the wind profile in the area seems to be the same at the measured point and at the analyzed location, as can be observed in Fig. 4, it was considered unnecessary to extrapolate the wind speed horizontally, but only vertically. The data were measured over the course of a year, from January 1, 2020, to December 31, 2020.

Assessing and analyzing wind energy resources is essential for the successful development of wind farm projects. Using meteorological data collected throughout 2020, this study undertakes a comprehensive evaluation of the wind energy potential within Romania's southeastern region.

**Figure 3:**
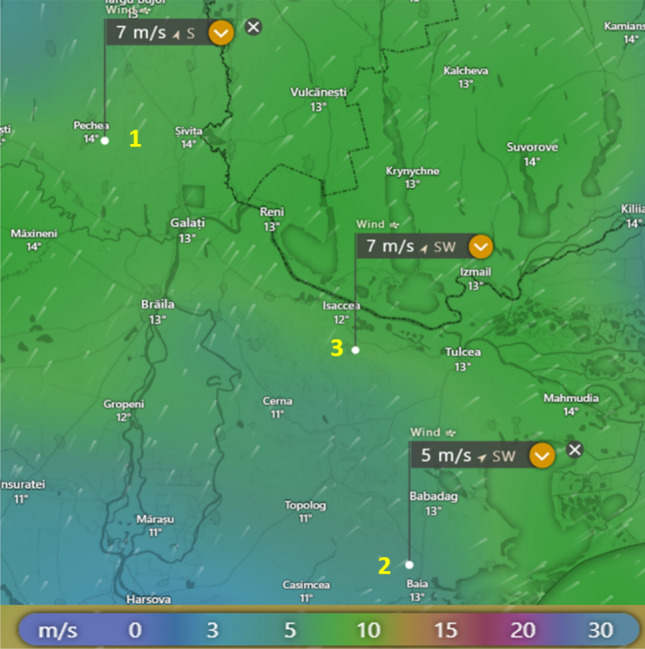
The analyzed location (source: windy.com)^29^.

**Figure 4:**
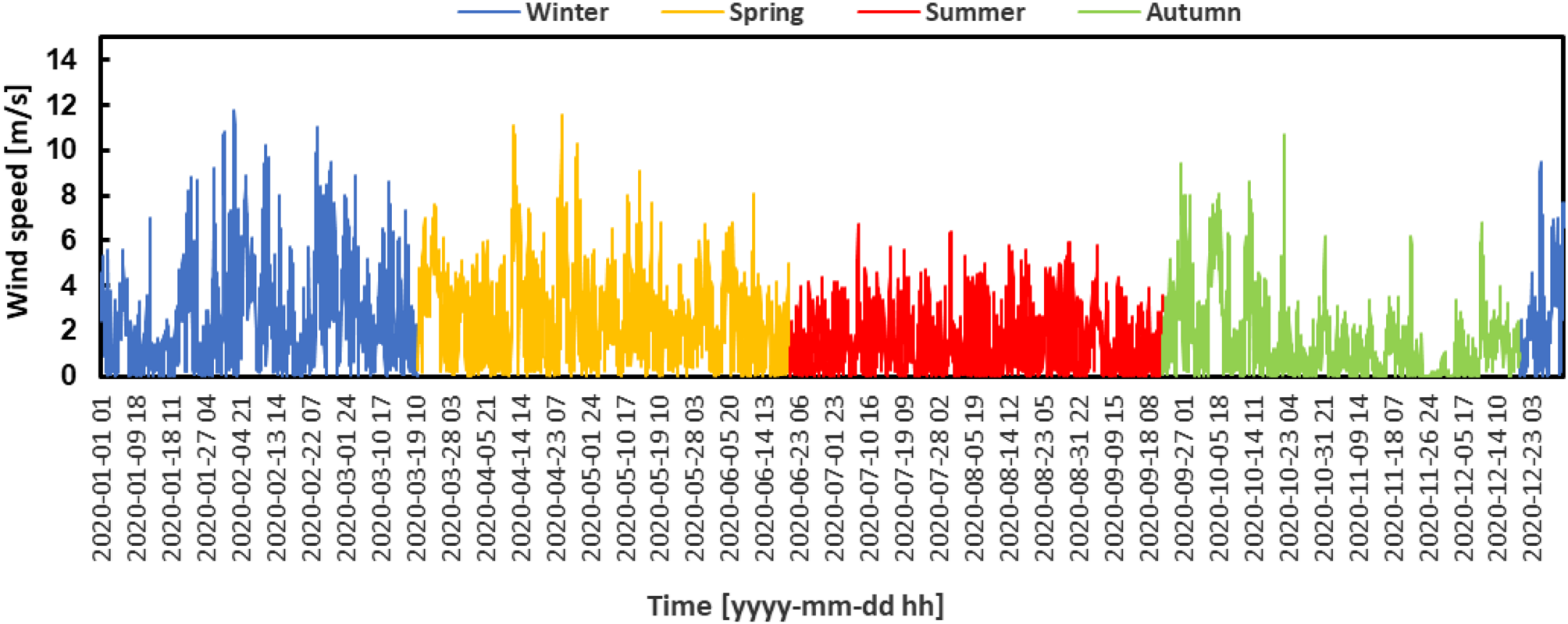
Hourly wind speed variation.

Location 3, illustrated in Fig. 3, is situated between two areas—locations 1 and 2—with high wind potential, where wind farms have been developed. One of these areas, location 2, hosts the Fantanele-Cogealac Wind Park, the largest onshore wind park in Europe, having a capacity of 600 MW. In this location, there are 2.5 MW wind turbines installed, while in location 1, the wind turbines have a power of 2 and 2.5 MW each. The purpose of the analysis is to evaluate the wind potential of location 3 and to determine the optimal power of the wind turbines that could be installed in this area. This approach is essential for the efficient and sustainable development of wind energy in the region (Fig. 4).

The dataset used in this analysis encompasses hourly average wind speed and direction measurements, collected at a standard height of 10 m above ground level. These parameters play a crucial role in evaluating the potential for wind energy capture in this region.

The analysis of wind energy potential was conducted using Python software, which can process a vast amount of data from CSV files^30^. Python, a versatile and powerful programming language, is widely used in data analysis and scientific computing due to its simplicity and the extensive availability of libraries. The analysis process involved reading the CSV files, which contained comprehensive wind speed and direction data, alongside other meteorological parameters. The utilization of Python's data manipulation packages, particularly Pandas, played a crucial role in arranging this data into well-organized formats, enabling easy and effective analysis. In this context, Python was employed to assess wind potential, a crucial step in developing renewable energy infrastructure. Python facilitates data segmentation by seasons, allowing a deeper understanding of seasonal variations in wind energy production. Thus, more accurate predictions can be made about turbine efficiency in different weather conditions.

Using this dataset, we compute various essential parameters that are fundamental to the planning of wind energy projects. This includes the total annual number of operating hours for the assessed wind turbines, which allows us to estimate their annual energy production. We also calculate the capacity factor for these turbines, which is an important indicator of their efficiency and performance.

This study provides insights into the wind energy potential in Romania's southeastern region by analyzing these parameters. It also offers essential insights for stakeholders and developers who aim to make well-informed decisions regarding wind farm investments in this region.

**Figure 5:**
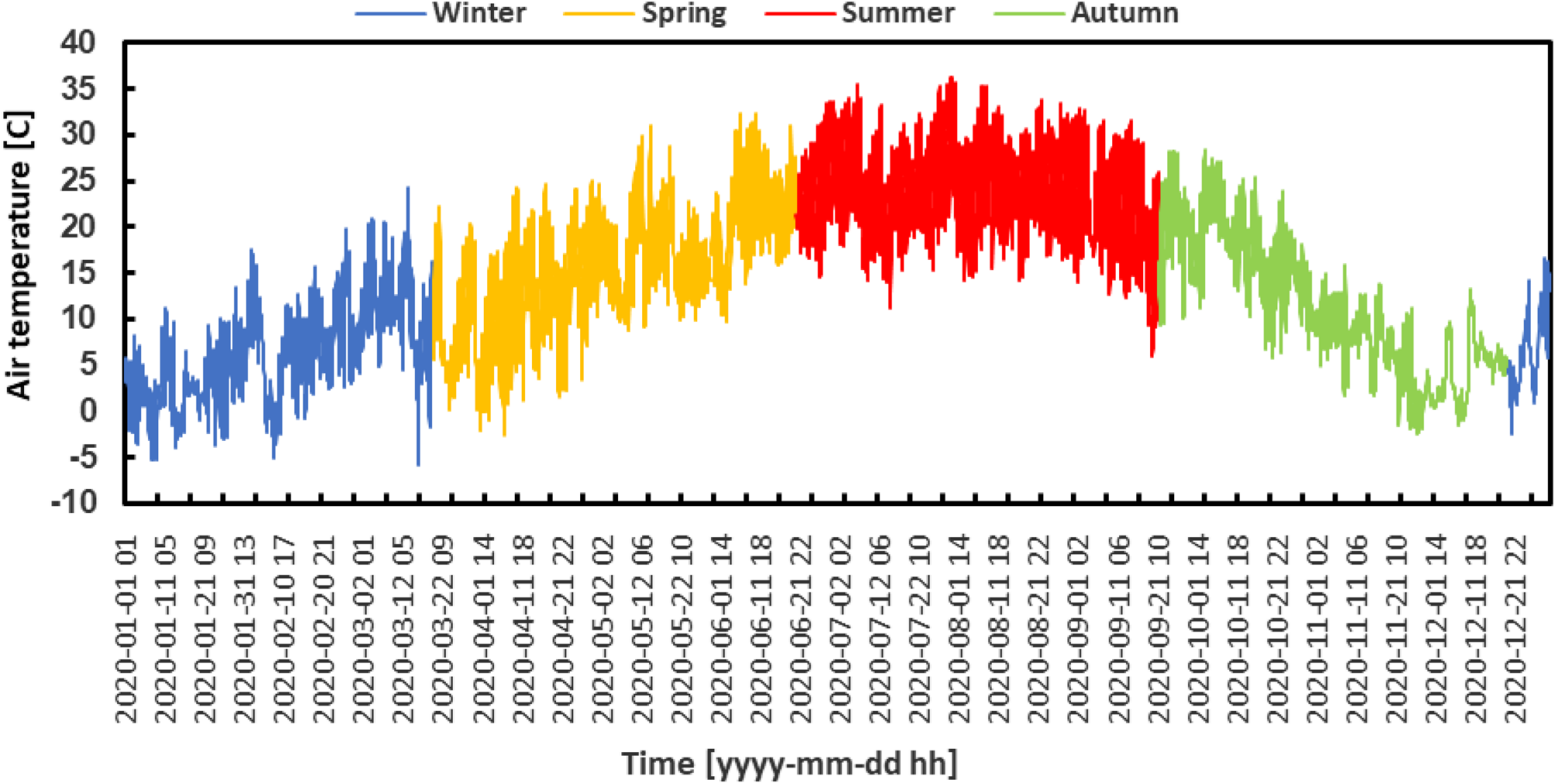
Hourly air temperature variation.

Figure 5 illustrates the diurnal temperature fluctuations observed on an hourly basis at the examined geographical site throughout the year 2020. The temperature data presented in this analysis demonstrate a year characterized by significant dynamism. On March 16th, the temperature reached a minimum of − 5.8 °C, marking the lowest recorded value. Conversely, on July 30th, the temperature peaked at 36.26 °C, making it as the highest recorded temperature of the year.

**Figure 6:**
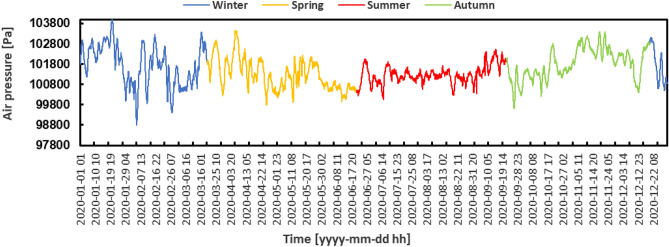
Hourly air pressure variation.

On the other hand, there were clear and obvious trends in atmospheric pressure, as shown in Fig. 6. During the summer months, there were few fluctuations observed, indicating a state of rather steady atmospheric conditions. Nevertheless, in the winter and spring seasons, there was a notable fluctuation in atmospheric pressure. On February 5th, the pressure dropped to its lowest point, measuring 987.9 Pa, while on January 10th, it peaked at 1040.9 Pa, demonstrating the extremes experienced in these seasons.

The notable fluctuations in temperature and pressure observed over the course of the year highlight the complex structure of the regional climate, making it an interesting subject needing further investigation and analysis.

**Figure 7:**
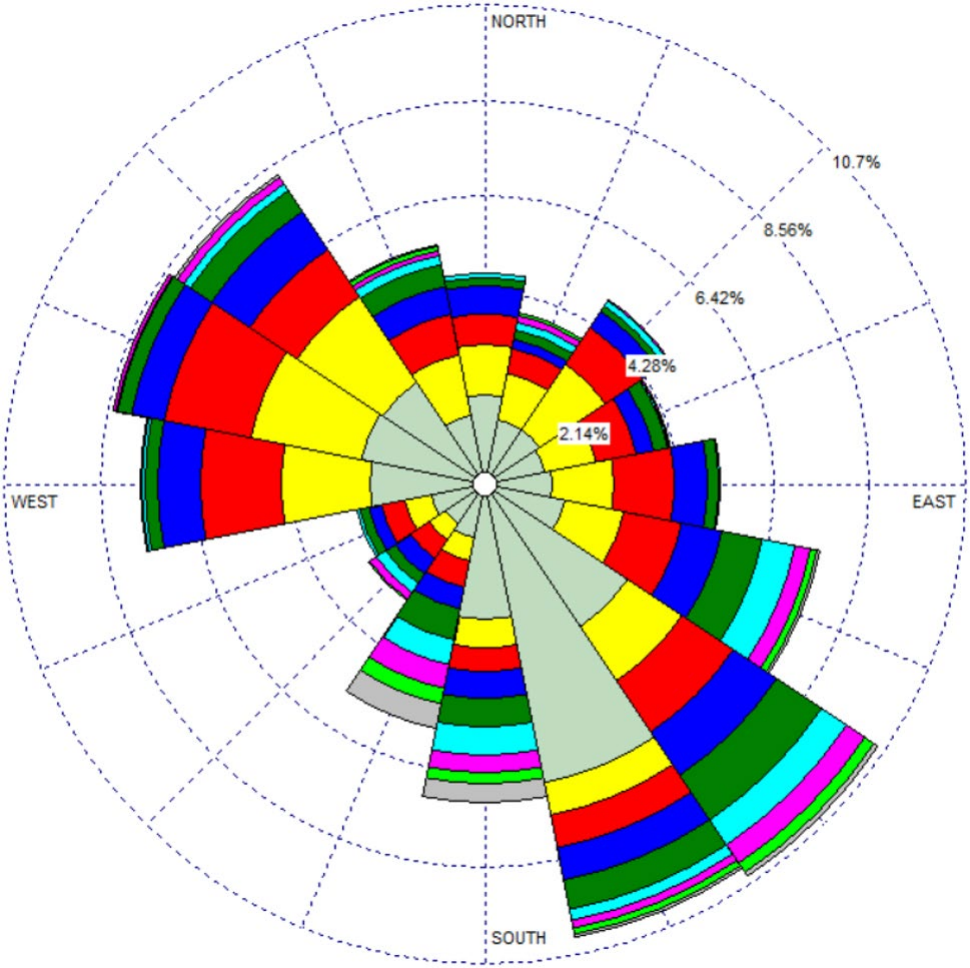
Wind rose at the analyzed location.

The wind rose for the region being analyzed is illustrated in Fig. 7. The prevailing wind direction was obtained by binning the wind directions into a wind rose with 16 segments. According to the graphic, the dominant wind directions observed are west-southwest (WSW) and northwest (NW), cumulatively having a frequency of approximately 25%. On the other hand, the data indicates that SE and SSE winds demonstrate a higher frequency, accounting approximately 35% of the total occurrences. The analysis of wind patterns presented in this study offers significant insights into the meteorological conditions specific to the local area.

### Probability density function

Several statistical distributions, including the commonly used Rayleigh and Weibull distributions, are essential for the characterization and analysis of wind resource data^31^. Among the various statistical methods available for modeling wind speed data, the Weibull distribution stands as an effective and reliable option^32,33^. The reason for its extensive implementation in the field of wind energy can be attributed to its capacity to accurately depict the characteristics of wind data^34^.

In this study, the wind speed potential at the selected location was evaluated using the Weibull probability density function. The statistical tool can be used to characterize the probability distribution of wind speeds. The probability density function of the measured wind speed was obtained by binning the data sets into 1 m/s wide intervals and calculation of data points percentage for each bin. The mathematical form of the Weibull distribution function is given by the following expression^35^:

1 $$f\left( v \right) = \left( \frac{k}{c} \right)\left( \frac{v}{c} \right)^{k - 1} exp\left[ { - \left( \frac{v}{c} \right)^{k} } \right]$$

In this context, the variable ‘v’ denotes the magnitude of wind speed, ‘k’ represents the shape parameter, and ‘c’ [m/s] represents the scale parameter associated with the Weibull distribution.

The form factor k and the scale factor c are established through Eqs. (2) and (3), respectively:

2 $$k = \left( {\frac{\sigma }{{\overline{v}}}} \right)^{ - 1.086}$$

3 $$c = \frac{{\overline{v}}}{{\Gamma \left( {1 + \frac{1}{k}} \right)}}$$

In the given expression, $\overline{v}$ represents the average wind speed, σ is the standard deviation of the wind speed, and Γ denotes the gamma function. The gamma function, often recognized as an extension of the factorial function to complex numbers, is formally expressed as:

4 $$\Gamma \left( x \right) = \mathop \smallint \limits_{0}^{\infty } t^{x - 1} e^{ - t} dt$$

Using this probability distribution to the recorded wind speed data, researchers can obtain significant knowledge about features of the wind resource^36,37^. This information is crucial for the effective planning and implementation of wind energy systems in the examined location. The utilization of this statistical methodology significantly improves our capacity to effectively exploit renewable energy sources, thereby making a substantial contribution to the adoption of sustainable energy practices.

### Wind turbine power curve

Wind speeds that can be used to generate energy are those that fall between the cut in wind speed and cut off wind speed. The power curve, which establishes a relationship between the power of the wind turbine and the wind speed, represents the power produced by the wind turbine at different wind speeds. The relationship between turbine power output and wind speed can be expressed mathematically as follows:

5 $$P_{WT} = \left\{ {\begin{array}{*{20}c} {0, v_{ci} > v_{i} > v_{co} } \\ {P\left( {v_{i} } \right), v_{ci} < v_{i} < v_{r} } \\ {P_{T} , v_{r} < v_{i} < v_{co} } \\ \end{array} } \right.$$

where vci, vr, and vco represent the cut in wind speed, nominal wind speed, and cut off wind speed for turbine protection, respectively.

The cut in wind speed of a wind turbine is the speed at which it begins to produce energy. If the wind speed is less than this, the turbine will not be able to produce electricity. When the wind speed is between the cut in wind speed and cut off wind speed, the wind turbine generates power according to the cubic relationship between wind speed and power. If the wind speed exceeds the maximum wind speed, the turbine is shut down.

**Figure 8:**
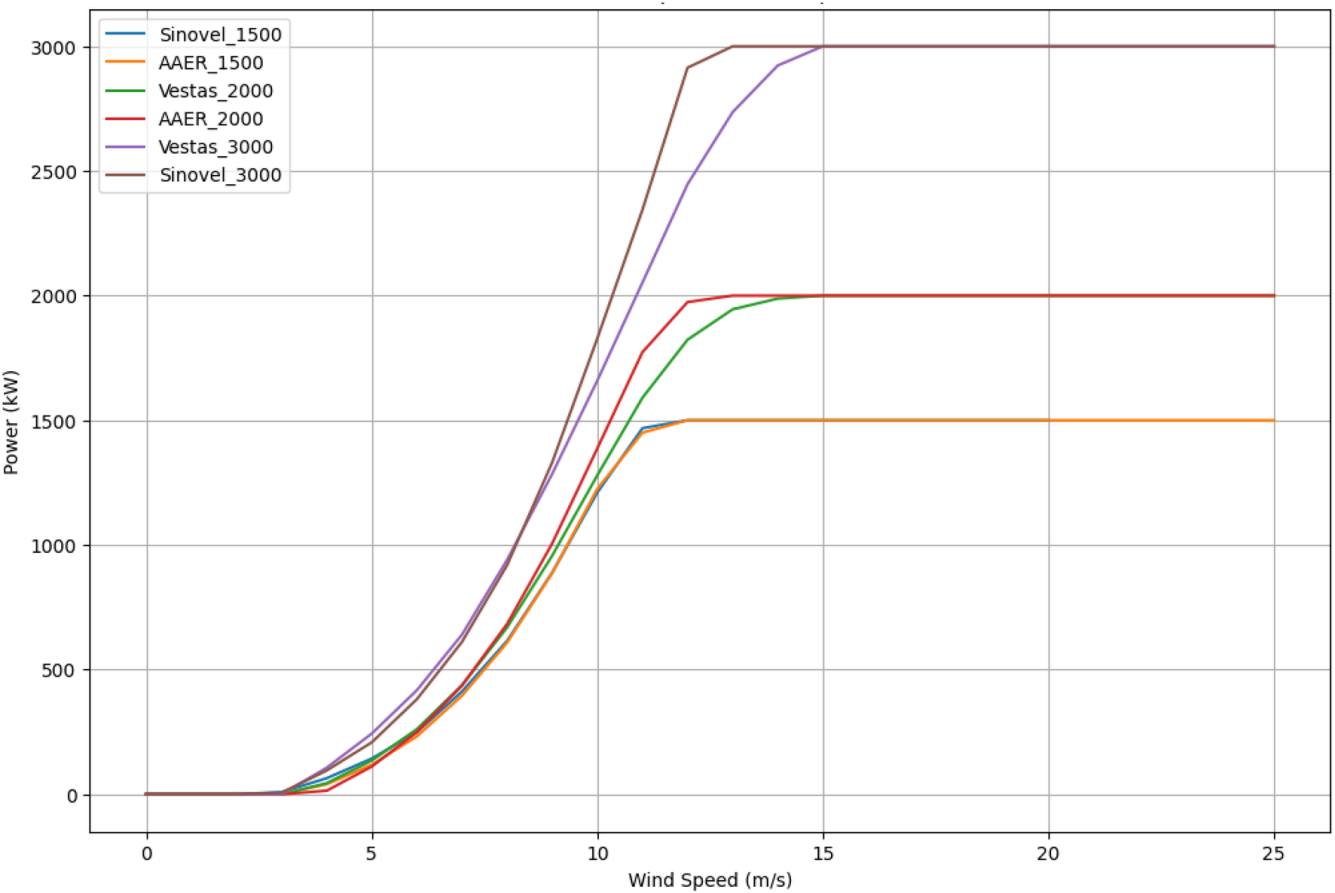
Power curves of wind turbines.

For the analysis, six types of turbines were studied for three power categories, namely 1.5, 2, and 3 MW. Figure 8 presents the power curves for the analyzed turbines, which are: Sinovel—1.5 MW, AAER—1.5 MW, Vestas—2.0 MW, AAER—2.0 MW, Vestas—3.0 MW, and Sinovel—3.0 MW.

The characteristics of the wind turbines examined are displayed in Table 1.

**Table 1:** Characteristics of wind turbines^38^.

	Sinovel SL1500/77	AAER A1500-70	Vestas V80/2000	AAER A2000-80	Vestas V90/3000	Sinovel SL3000/90
Cut-in wind speed (m/s)	3.5	4	3	4.5	4	4.5
Rated wind speed (m/s)	11	12	13.5	12	14	12
Cut-off wind speed (m/s)	20	20	25	25	25	25
Rated power (kW)	1500	1500	2000	2000	3000	3000
Rotor diameter (m)	77.4	70	80	80	90	91.6
Hub height (m)	80	80	78	80	80	80

### Vertical distribution of wind speed

Since wind speed measurement was not conducted at the operating height of the wind turbines, it is necessary to extrapolate the wind speed values to the height of the hub, which is also the height at which turbine manufacturers provide the power curves for these turbines. Since wind speed increases with altitude, the power law model was used to extrapolate the wind speed to the hub height of location according to the vertical wind profile relationship^39^.

6 $$v = v_{0} \left( {\frac{h}{{h_{0} }}} \right)^{n}$$

where v: wind speed at the turbine hub height (m/s). h: turbine hub height (m). v_0_: wind speed measured at the anemometer height (m/s). h_0_: height of the anemometer (m). n: wind shear coefficient.

### Assessment of wind power potential

The concept of wind power potential refers to the theoretically available amount of wind power at the specific location. The first step in estimating the power potential of a wind site involves collecting wind data because the intermittency and variability of the wind make it challenging to predict its power potential accurately^40^. Despite all the analyses and modeling conducted to date, a precise estimation of wind potential at any location globally has not been achieved. Modeling the wind speed distribution involves fitting a known continuous function (such as Weibull) to match the histogram of collected wind speed data at the analyzed location. The available power from the wind, P_W_, is given by 7^41^:

7 $$P_{W} = \frac{1}{2}\rho_{a} A\mathop \smallint \limits_{0}^{\infty } f\left( v \right)v^{3} dv$$

where $\rho_{a}$ is air density, $f\left( v \right)$ is probability density function, variable A denotes the spatial area swept by the blades, and v represents the instantaneous wind speed.

The variation of air density in the troposphere as a function of altitude was determined by considering the variation of temperature and pressure with altitude^42^.

Pressure variation as a function of height was calculated with Eq. 8:

8 $$P = P_{b} \left[ {\frac{{T_{b} - \left( {h - h_{0} } \right)T_{lr} }}{{T_{b} }}} \right]^{{\frac{gM}{{RT_{lr} }}}}$$

where $P_{b}$, barometric pressure (N/m^2^). $T_{b}$, measured temperature (K). $T_{lr}$, temperature lapse rate 0.0065 (K/m). *h*, interested height (m). $h_{0}$, reference height (m). R = universal gas constant: 8314.4598 J/(kmol·K). g, gravitational acceleration: 9.81 m/s^2^. M, molar mass of Earth's air: 28.9644 kg/kmol.

The variation of temperature with altitude based on the lapse rate was calculated using Eq. (9):

9 $$T = T_{b} - h \cdot T_{lr}$$

The equation for computing the variation of density as a function of height in the troposphere, considering changes in relative pressure and temperature changes, is:

10 $$\rho_{a} = \rho_{b} \frac{P}{T}\frac{{T_{b} }}{{P_{b} }}$$

where $\rho_{b} -$ air mass density at sea level 1.225 kg/m^3^.

The available wind energy Ea for a time of N hours is given by the relation 11:

11 $$E_{a} = \frac{{P_{W} }}{1000}N$$

The recoverable energy at the investigated site, based on the power curve characteristics of the wind turbine, is determined with the relation 12^43^:

12 $$E_{er} = \frac{N}{1000}\frac{1}{2}\rho_{a} A\left[ {\mathop \smallint \limits_{{V_{c\_in} }}^{{V_{r} }} f\left( v \right)v^{3} dv + \mathop \smallint \limits_{{V_{r} }}^{{V_{c\_out} }} f\left( v \right)V_{r}^{3} dv} \right]$$

where V_c_in_ is the cut-in speed of wind turbine; V_r_ is the rated wind speed of wind turbine; V_c_out_ is the cut-out wind speed of wind turbine.

The capacity factor allows us to determine the most suitable turbine for installation at the analyzed location, as follows:

13 $$CF = \frac{{E_{er} }}{{E_{i} }}$$

The maximum annual energy Ei, that can be generated by the wind turbine is given by the relationship (14):

14 $$E_{i} = 8760 E_{W}$$

E_w_—rated turbine power, (kW).

Carbon emission savings are calculated using the emission factor, $e_{CO2}$, for the national electricity generation system. The annual emission savings, denoted as V_CO2_ (t CO_2_/year), are calculated by multiplying the total annual energy output of the wind turbine, represented as E_er_ (MWh/year), by the emission factor $e_{CO2}$ (t CO_2_/MWh) for the reference scenario of the national electricity production system ^44^.

15 $$V_{CO2} = E_{er} \cdot e_{CO2}$$

## Results and discussions

### Analysis of wind data

**Figure 9:**
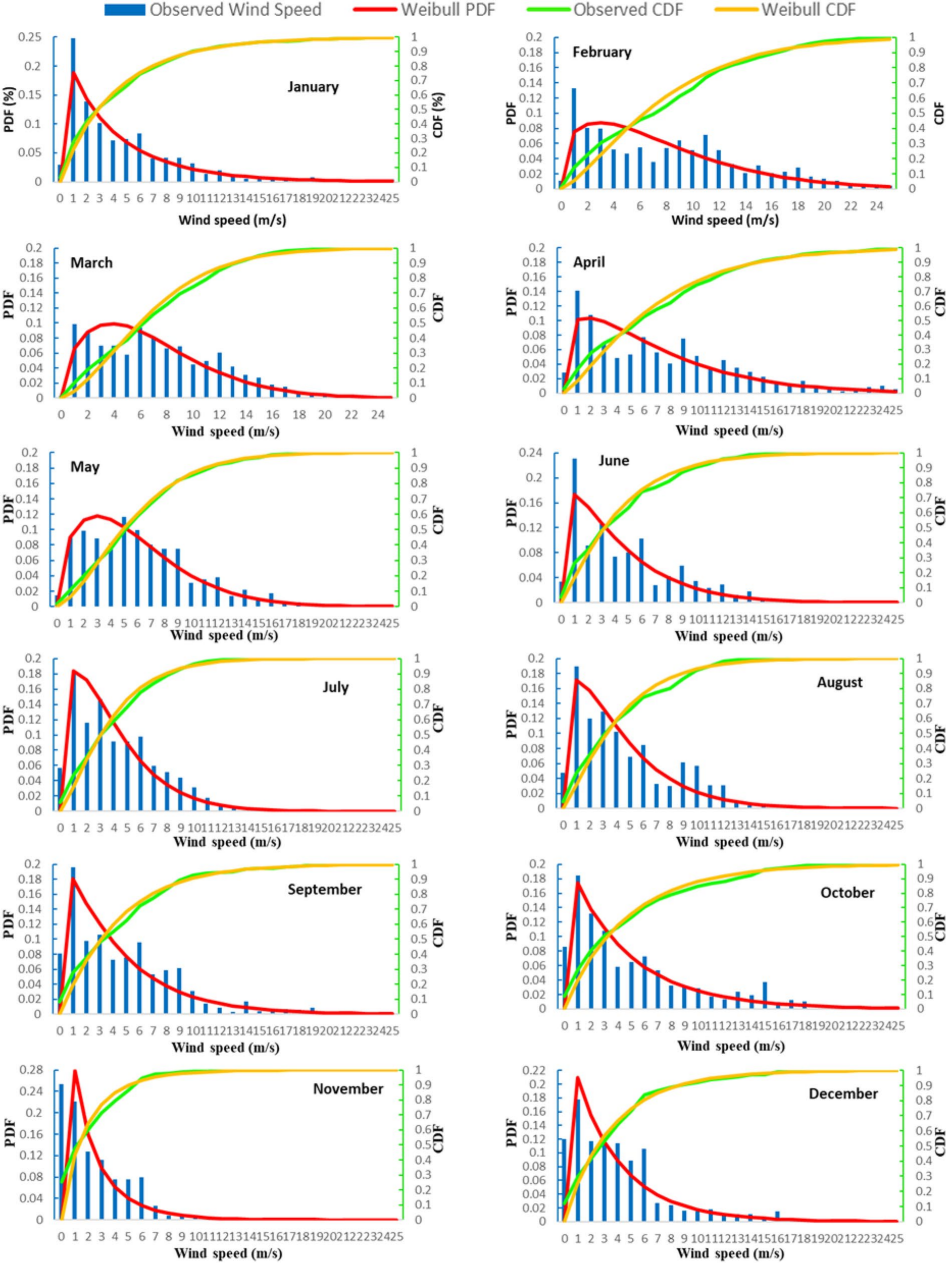
Monthly variation of the Weibull probability density function and the cumulative distribution function at the analyzed location.

The Weibull distribution is determined by two parameters, c and k, which are the scale and shape parameters, respectively. Figure 9 shows the monthly PDF curves. We observed that if k increases, the Weibull density function becomes narrower and larger. In addition, if k increases, the tip of the curve shifts to the right. If the period is not too short, the meteorological data collected can be reasonably described by the Weibull density function.

Based on hourly meteorological data measured in 2020, Weibull parameters (“k” shape coefficient and “c” scale coefficient) that describe the profile of the Weibull distribution curve were calculated.

The “k” shape parameter was determined to be between 0.86 and 1.53 for the hub's 80 m height, with a minimum in November and a maximum in March. Meanwhile, the Weibull scale parameter "c" ranged between 1.92 and 8.37 m/s, with a minimum in November and a maximum in February. Figure 11 depicts the monthly variation of these parameters. It can also be seen that as the wind speed increases, the value of the scale parameter does as well.

According to Fig. 9, the cumulative frequency varies between 21 and 67% for wind speeds greater than 4 m/s at a height of 80 m. Furthermore, the cumulative frequency indicated that the likelihood of higher wind speeds exceeding 20 m/s was reduced in 2020. The probability of wind speed between 4 and 20 m/s for this site is quite high, as this is the typical operating range of the most of wind turbines analyzed. The turbines considered in the study have a cut in wind speed of 3.5–4.5 m/s and a nominal speed of 10.5–15 m/s.

**Figure 10:**
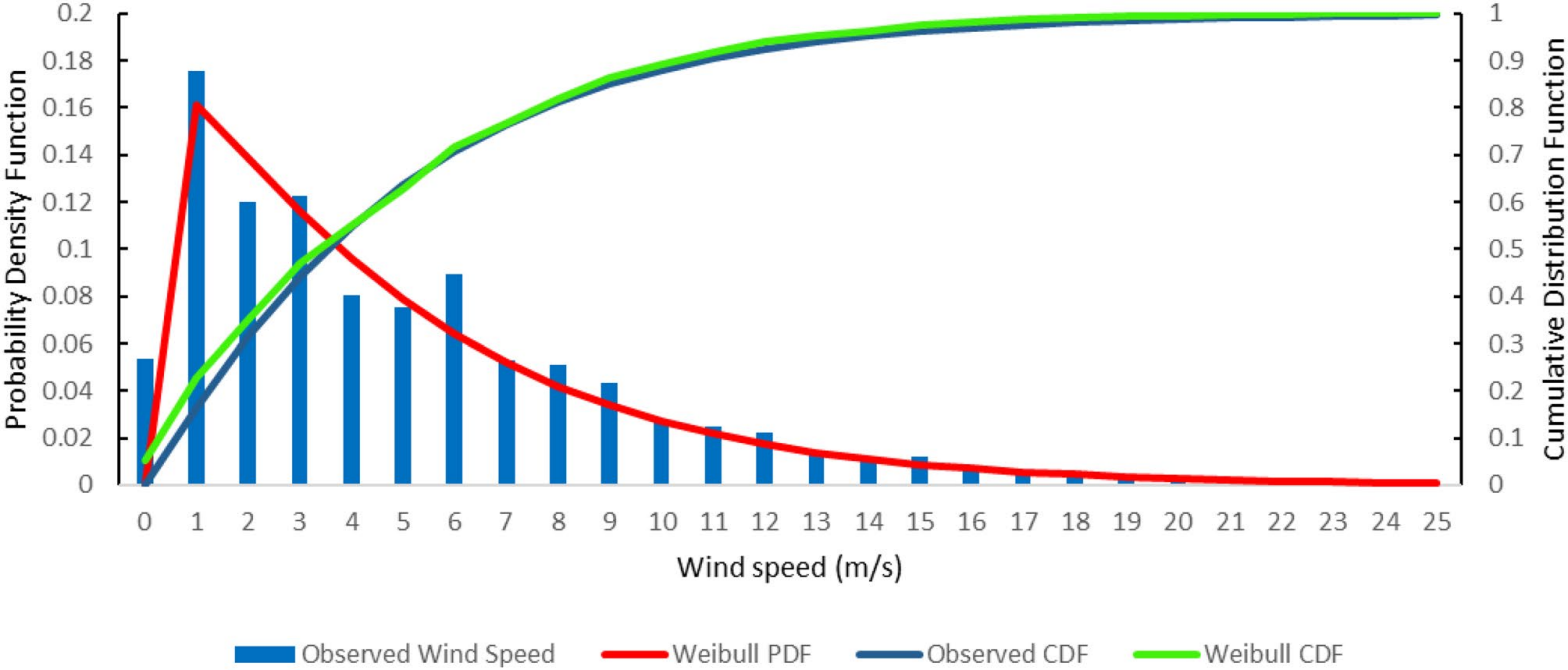
Annual variation of the Weibull probability density function and the cumulative distribution function.

**Figure 11:**
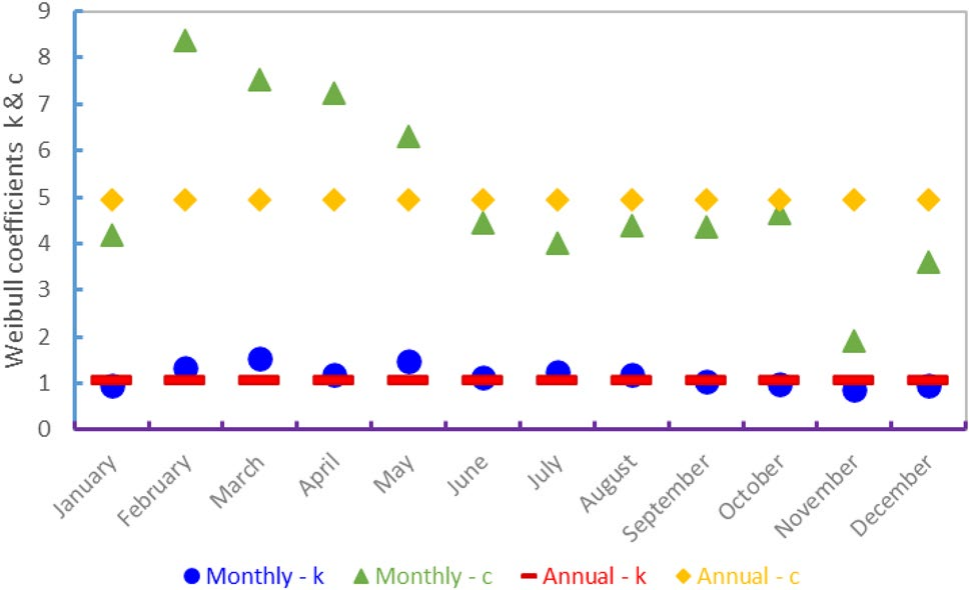
Monthly and annual variation of Weibull coefficients.

Short periods of time, such as an hour or a day, are not well-described by a Weibull or any other statistical function, but for longer periods of time, such as several weeks to a year or more, Weibull generally matches the observed data reasonably well. Figure 10 presents the annual PDF and CDF functions. For the annual variation, the Weibull coefficients c and k were 4.95 and 1.07. When k is greater than the unit, PDF equals zero at zero wind speeds. As a result, the Weibull density function cannot adapt a zero-speed wind speed curve. This is not a serious issue for wind power applications because the output of a wind turbine would be zero at a wind speed lower than the turbine cut in wind speed. As shown in Fig. 10, the Weibull distribution accurately describes wind speed on an annual basis (Fig. 11).

The Wind Power Density (WPD) was determined by measuring wind speed at the analyzed location and considering the air density. Wind speed data collected from the meteorological station at a height of 10 m was extrapolated to the turbine hub height (80 m) using the power law to account for altitude variations in wind speed. Air density, influenced by altitude, temperature, and pressure, was determined using the ideal gas law and standard values. The formula for wind energy density derives from the principles of kinetic energy and considers the cubic impact of wind speed. WPD was then used to estimate energy production at the analyzed location.

**Figure 12:**
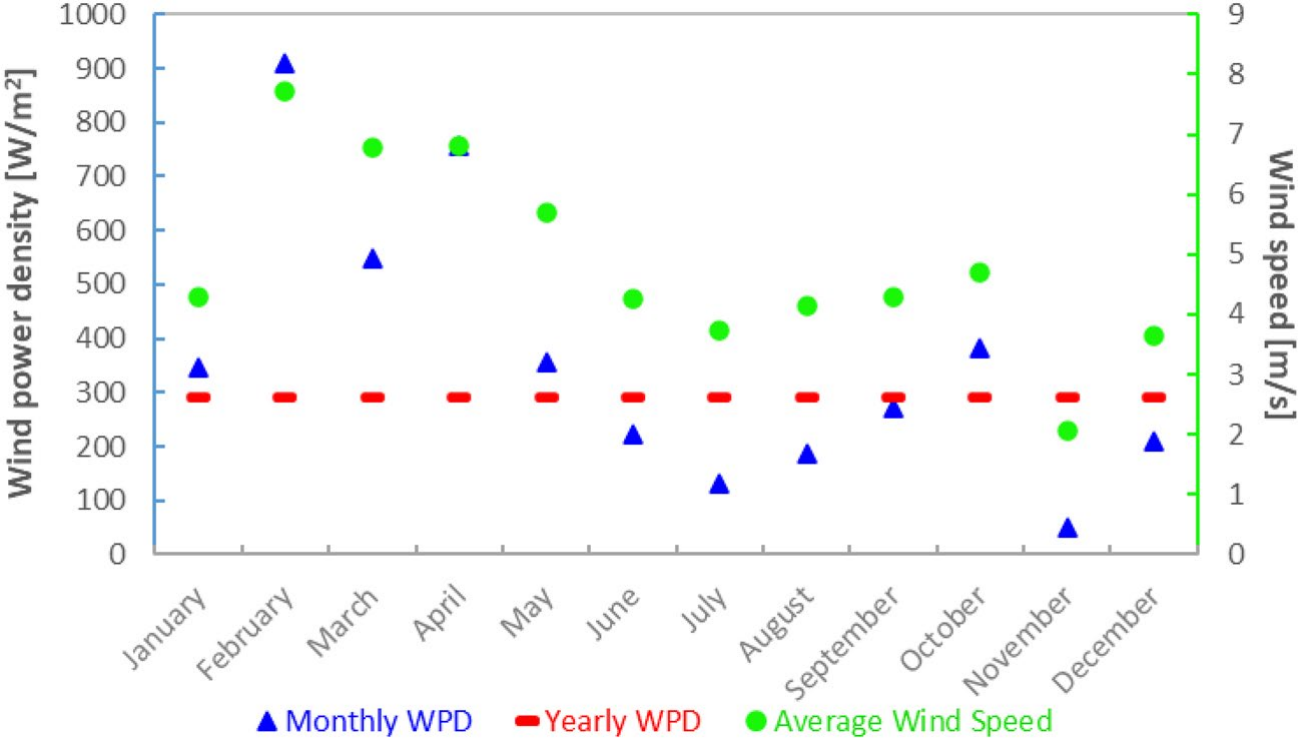
Monthly and annual variation of wind power density and monthly average wind speed variation.

According to Fig. 12, the average monthly wind speed in the analyzed location ranged between 2.07 and 7.71 m/s, and the wind power density ranged between 50 and 910 W/m^2^ with an annual value of 290 W/m^2^.

It was observed that for the same average wind speed of 4.29 m/s in January and September, the value of WPD varies greatly, being 347 and 273 W/m^2^ respectively, indicating that the approach of WPD analysis based on average wind speed in a location can lead to significant errors, with the variation in this case being around 21%.

### Energy production

**Figure 13:**
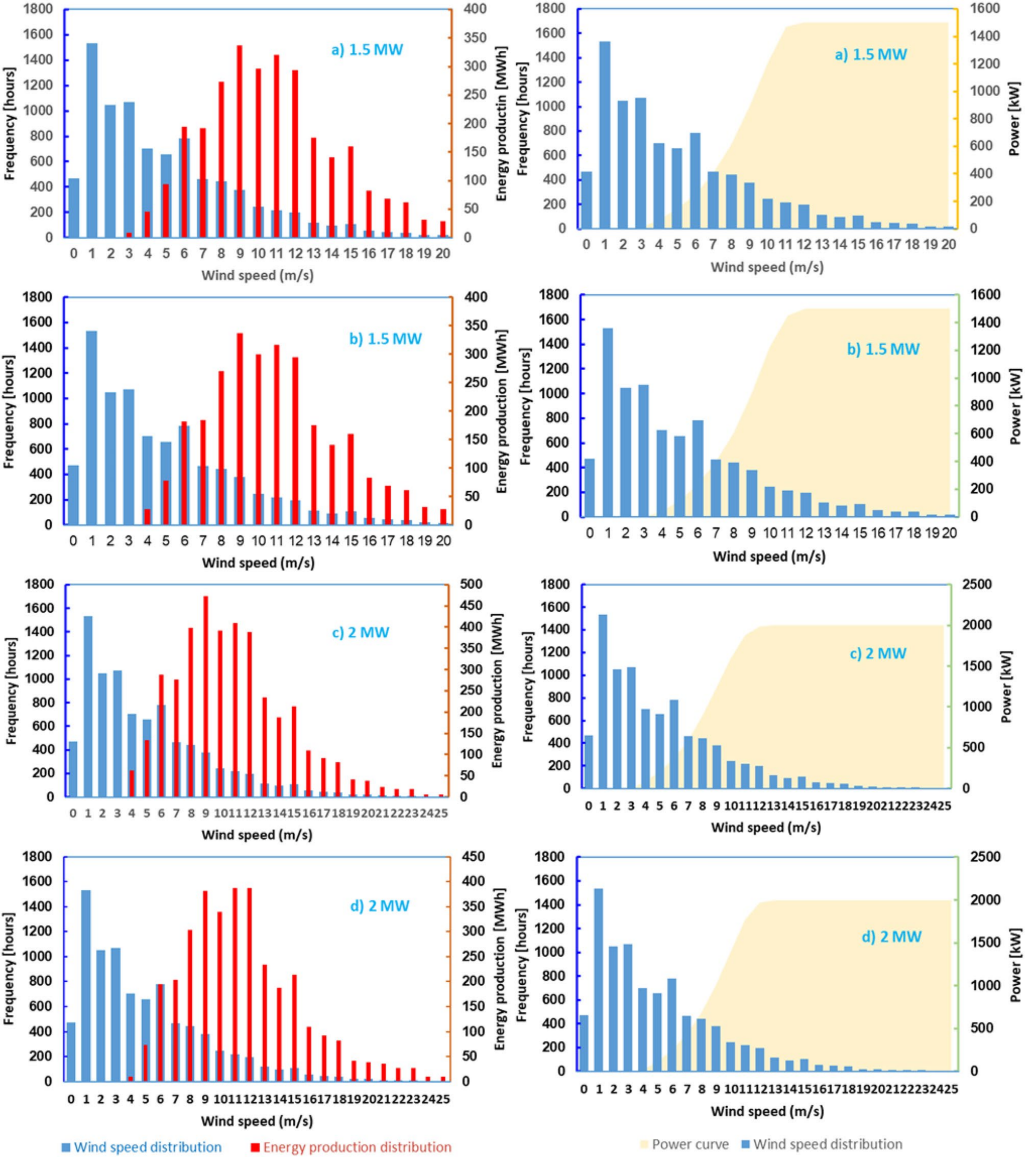
Power curves and energy produced by turbines for various wind speed frequencies: (**a**) Sinovel—1.5 MW, (**b**) AAER—1.5 MW, (**c**) Vestas—2.0 MW, (**d**) AAER—2.0 MW.

**Figure 14:**
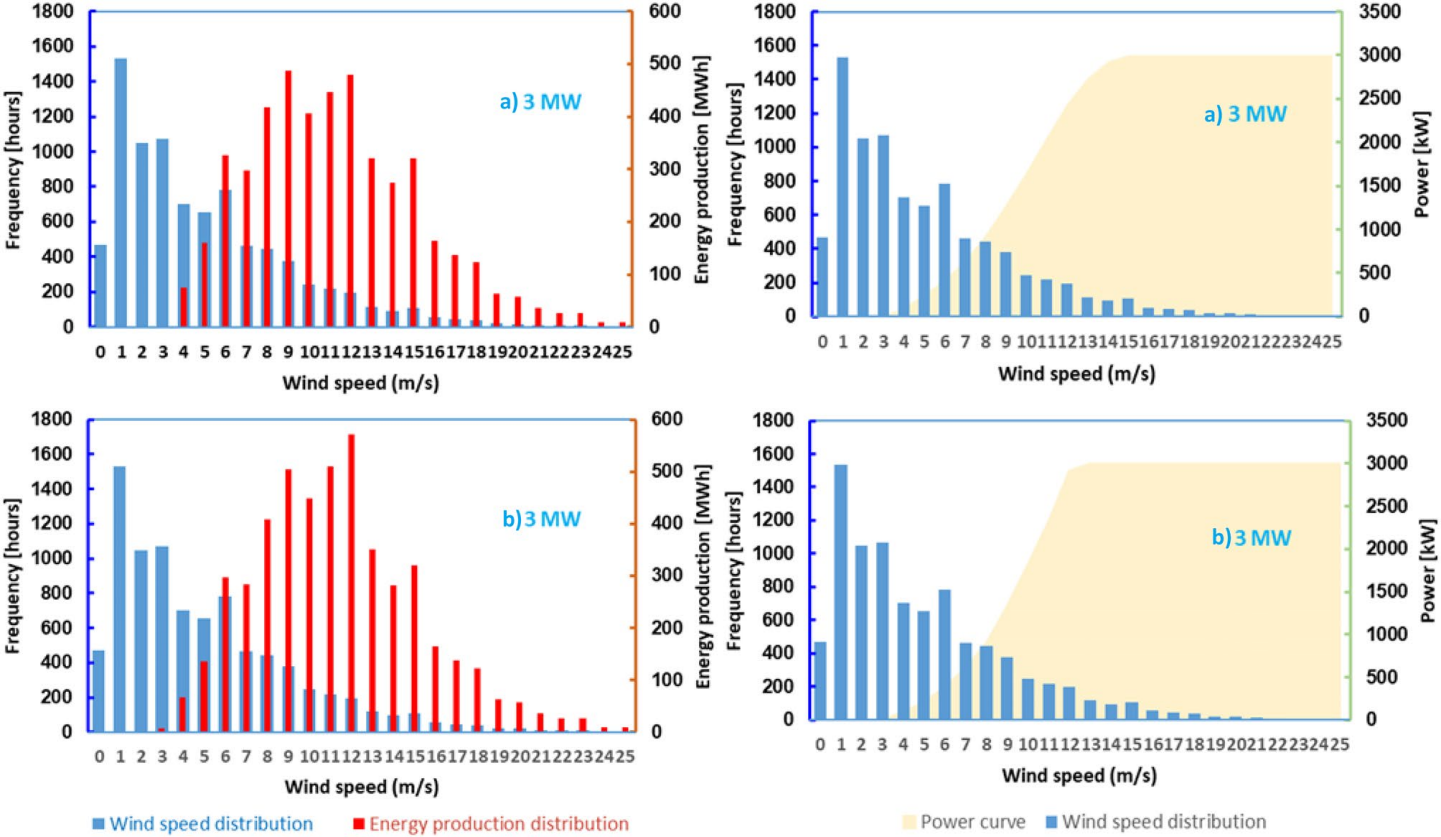
Power curves and energy produced by turbines for various wind speed frequencies:—(**a**) Vestas—3.0 MW, (**b**) Sinovel—3.0 MW.

Figures 13 and 14 presents the distribution of energy production as well as the power curves for the turbines under consideration. According to data analysis, the Vestas 3.0 MW turbine reaches its maximum power at a wind speed of 15 m/s, whereas the Vestas 2.0 MW turbine reaches its maximum power at a wind speed of 13 m/s, allowing it to achieve higher performance.

The number of hours available annually for the analyzed turbines when the wind speed is strong enough for the wind turbines to operate at nominal power is between 536 and 950 h, and the total number of operating hours for speeds between cuts-in and cut-out is between 4624 and 5694.

### Capacity factor and avoided CO_2_ emissions

Wind turbines performance is essential for the development of wind energy project at the location. The selection of a wind turbine suitable for the conditions of the wind potential from the analyzed location is very important because this process will influence the energy production and respectively the objective of obtaining maximum efficiency. Wind turbines' annual energy production for this location was analyzed based on their capacity factor which refers to the ratio of the actual output of the wind turbines to their maximum potential output.

The value of the capacity factor is influenced by the sporadic nature of the wind speed, the number of hours the wind turbines are operational, and their efficiency.

**Figure 15:**
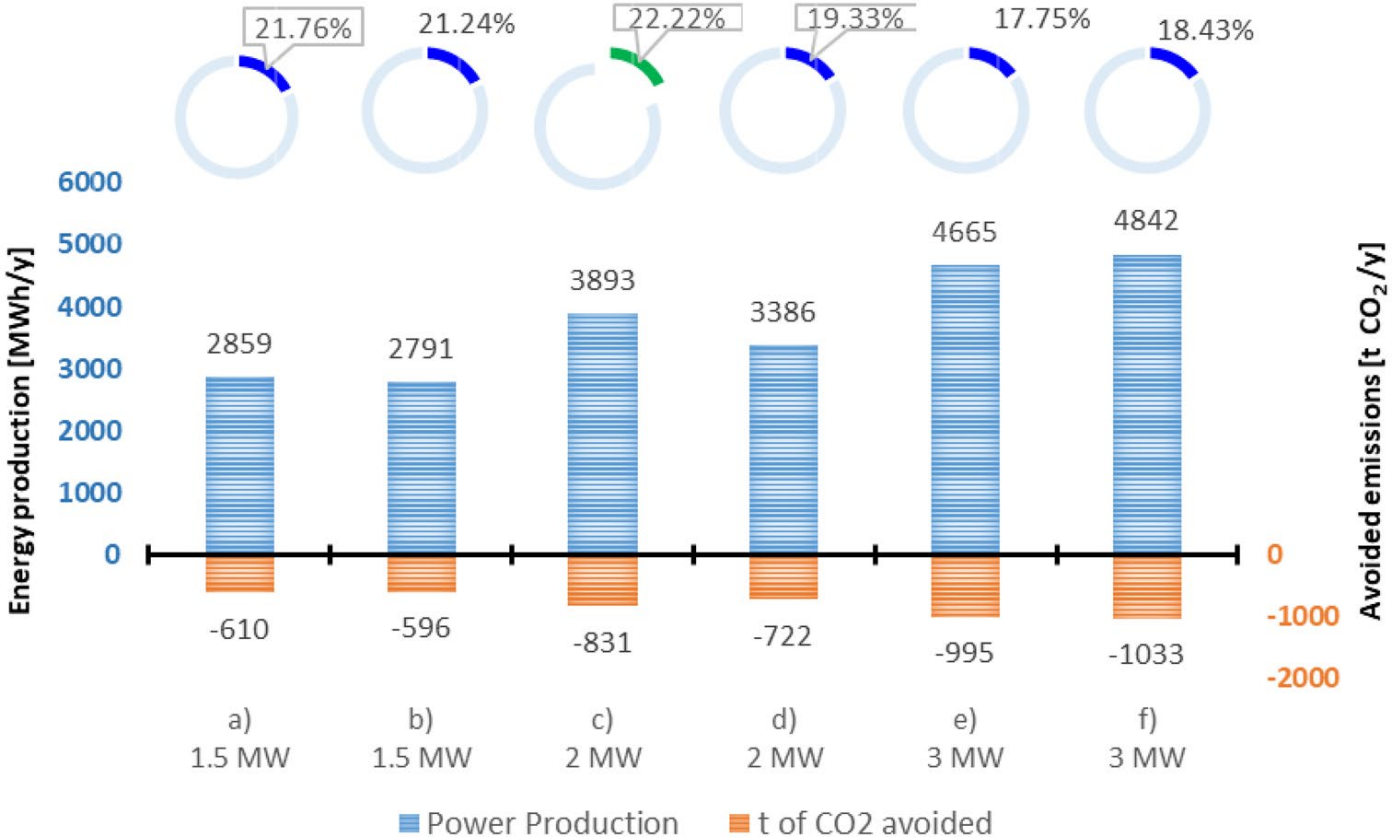
Energy production, avoided emissions, and capacity factor for the turbines investigated: (**a**) Sinovel—1.5 MW, (**b**) AAER—1.5 MW, (**c**) Vestas—2.0 MW, (**d**) AAER—2.0 MW, (**e**) Vestas—3.0 MW, (**f**) Sinovel—3.0 MW.

The energy production and capacity factor of six different wind turbines with nominal powers ranging from 1.5 MW to 3.0 MW were determined and presented in Fig. 15. The annual energy production was calculated using the power curve of each wind turbine and the number of operating hours calculated for each speed range.

Figure 15 illustrates the annual energy produced and the value of the capacity factor for all the wind turbines studied. The maximum annual energy production of 4842 MWh is achieved for a capacity factor of 18.43% of the Sinovel wind turbine − 3.0 MW, as shown in Fig. 15. The minimum annual energy production was obtained as 2791 MWh, but for a capacity factor of 21.24% of the AAER turbine − 1.5 MW. The annual energy generated by the six wind turbines was estimated to be between 2791 and 4842 MWh, as shown in Fig. 15.

This figure also shows the annual amount of emissions avoided due to the use of renewable energy sources respectively through the operation of the wind turbines that are being investigated. The avoided emissions are expressed in tons of CO_2_ equivalent, and an emission index of 213.37 g CO_2_/kWh specific to the Romanian electricity network in 2020 was used for their calculation^45^. Annual emissions not released due to wind turbine electricity production were calculated to be between 596 and 1033 t CO_2_ equivalent.

## Conclusions

Electricity prices have risen dramatically in recent years because of policies aimed at limiting global average temperature rise to a maximum of 1.5 °C. As a result, there is an increased need to generate electricity from renewable sources, and wind energy is a source capable to produce electricity at affordable prices.

To analyze which type of wind turbine best fits the wind resources of the investigated location in terms of power, this paper has reviewed and compared six wind turbines with three different powers, namely 1.5, 2, and 3 MW, from three different manufacturers: Sinovel, AAER, and Vestas. The comparative analysis of the wind turbines regarding wind speed, turbine energy production, and capacity factor showed that the most suitable wind turbine for installation at the location is the one with a nominal power of 2 MW. The analyzed location is situated between two areas with high wind potential, where wind parks composed of wind turbines with powers ranging from 2 to 2.5 MW in the north and 2.5 MW in the south are built.

The wind characteristics and wind energy potential of an onshore location in southeastern Romania were examined in this study. At the analyzed location, the average monthly wind power density ranges from 50 to 910 W/m^2^ for a height of 80 m, while the Weibull shape and scale parameters range from 0.86 to 1.53 and 1.92 to 8.37 m/s, respectively.

The calculated energy production for six different types of commercially available wind turbines with powers ranging from 1.5 to 3.0 MW is in the range of 2791–4842 MWh per year, with a capacity factor ranging from 17.75 to 22.22%. The Vestas wind turbine − 2 MW has the highest capacity factor of 22.22%, with an estimated maximum annual energy production of 3893 MWh.

The wind turbine with the highest power production was Sinovel 3.0 MW, which produced 4842 MWh but had a capacity factor of only 18.43%.

Even if the energy production generated by the Sinovel 3.0 MW turbine was the highest, the analysis of the capacity factor shows that it operates at maximum capacity for only a very short period of the year, and therefore the power of this turbine is oversized for the wind potential in the analyzed area.

The evaluation of the wind energy potential of the analyzed site concluded that the most suitable turbines for power generation are those with a power of 2 MW, which also have the higher capacity factor.

Although the analyzed site does not have the same elevation as the adjacent locations, it can be observed that this lower level still creates a sufficiently intense air flow, making this location suitable for installing wind turbines to exploit the area's wind potential.

## Data Availability

The datasets used and analyzed during the current study are available from the corresponding author on reasonable request.

## List of symbols

A: The spatial area swept by the blades (m^2^)
c: The scale parameter (m/s)
${\text{CF}}$: The capacity factor
Ea: The available wind energy
E_er_: Annual energy output of wind turbine (MWh/year)
Ei: The maximum annual energy production
E_w_: Rated turbine power (kW)
${\text{e}}_{{{\text{CO}}2}}$: Emission factor of national electricity generation system, (t CO_2_/MWh)
$f\left( v \right)$: Probability density function
g: Gravitational acceleration (m/s^2^)
h: Turbine hub height (m)
h_0_: Height of the anemometer (m)
k: The shape parameter
M: Molar mass of air (kg/kmol)
N: Hours
n: Wind shear coefficient
$${\text{P}}_{{\text{b}}}$$: Barometric pressure (N/m^2^)
P_W_: The available power from the wind
R: Universal gas constant (J/(kmol K))
$${\text{T}}_{{\text{b}}}$$: Measured temperature (K)
${\text{T}}_{{{\text{lr}}}}$: Temperature lapse rate (K/m)
V: Wind speed at the turbine hub height (m/s)
V_CO2_: Annual emission savings (t CO_2_/year)
V_c_in_: Is the cut-in speed of wind turbine
V_c_out_: The cut-out wind speed of wind turbine
V_r_: The rated wind speed of wind turbine
$\overline{v}$: The average wind speed (m/s)
v_0_: Wind speed measured at the anemometer height (m/s)
$\rho_{a}$: Air density (kg/m^3^)
$\rho_{b}$: Air mass density at sea level (kg/m^3^)
Γ: The gamma function
σ: The standard deviation of the wind speed
